# Beyond pills and prescriptions: Exploring aging-related diseases with non-pharmacological strategies and molecular innovations

**DOI:** 10.14336/AD.2024.0560

**Published:** 2025-02-20

**Authors:** Mahdi Esmaeilzadeh, Nasrollah Moradikor

**Affiliations:** ^1^Department of Cell and Molecular Biology, International Center for Neuroscience Research, Institute for Intelligent Research, Tbilisi, Georgia.; ^2^Scientific Research Publishing House (SRPH), Shirvan, Iran.; ^3^International Center for Neuroscience Research, Institute for Intelligent Research, Tbilisi, Georgia.

**Keywords:** Aging-related diseases, molecular mechanisms, non-pharmacological interventions, innovative therapeutic approaches, precision medicine, gene therapy, challenges, future directions

## Abstract

This paper provides a thorough examination of aging-related diseases, exploring into the intricate molecular mechanisms that underline their development and progression. It explores cutting-edge therapeutic interventions aimed at addressing these conditions, with a particular focus on non-pharmacological approaches such as personalized lifestyle modifications, cognitive enhancement strategies, and robust social engagement initiatives. Additionally, it highlights emerging modalities including gene therapy and precision medicine as promising avenues for mitigating the challenges associated with age-related ailments. Despite significant strides in research, persistent barriers such as limited healthcare access and regulatory complexities continue to impede the widespread implementation of these innovative approaches. Overcoming these obstacles requires collaborative efforts to promote health equity and ensure equitable access to transformative treatments. By advancing our understanding and embracing innovative paradigms, we can catalyze a profound transformation toward healthier aging trajectories. Through sustained interdisciplinary collaborations, rigorous research endeavors, and proactive advocacy initiatives, we can aspire to cultivate a future where aging is synonymous with vitality, dignity, and enhanced well-being for individuals worldwide.

## 1. Introduction

The expanding prevalence of aging-related ailments poses a significant challenge in contemporary healthcare, warranting a comprehensive understanding and proactive management. With advancing age, individuals demonstrate an increased susceptibility to a selection of health adversities, spanning neurological, cardiovascular, metabolic, and immune perturbations [1]. These conditions not only compromise the qualitative aspect of life but also impose substantial burdens on healthcare infrastructures globally, necessitating innovative modalities for intervention. The salience of revealing aging-related diseases resides in their intricate connection with the aging process per se. Aging embodies not a mere chronological occurrence, but a multifaceted physiological regression marked by progressive decay in tissue and cellular function [2]. Solving the complexities underpinning aging stands as a pivotal prerequisite for pinpointing therapeutic targets and formulating interventions to alleviate the ramifications of age-related maladies.

In this context, this paper serves as a crucial avenue for exploring the multifaceted panorama of aging-associated afflictions. Through a fusion of research findings, scholarly critiques, and expert insights, the paper endeavors to propel our comprehension of the molecular nuances propelling aging while exploring innovative pathways for intervention [3-5].

This paper seeks to transcend the traditional belief on pharmaceutical remedies and dive into the realm of non-pharmacological schemes and molecular innovations in combatting aging-related diseases. By broadening our purview and embracing diverse modalities of intervention, we stand to unearth novel trajectories for fostering healthy aging and prolonging longevity. This paper lays the groundwork for navigating the intricacies of aging-related diseases, underscoring the pivotal role of non-pharmacological interventions and molecular innovations in shaping the trajectory of healthcare. In the ensuing sections, we will delve into the main themes of this paper, scrutinizing the evolving landscape of aging research and the potential it harbors for addressing the challenges engendered by aging-related diseases.

## 2. Non-pharmacological Therapeutic Approaches in Aging and Age-Associated Diseases

### 2.1. Lifestyle Interventions

**Figure 1.**
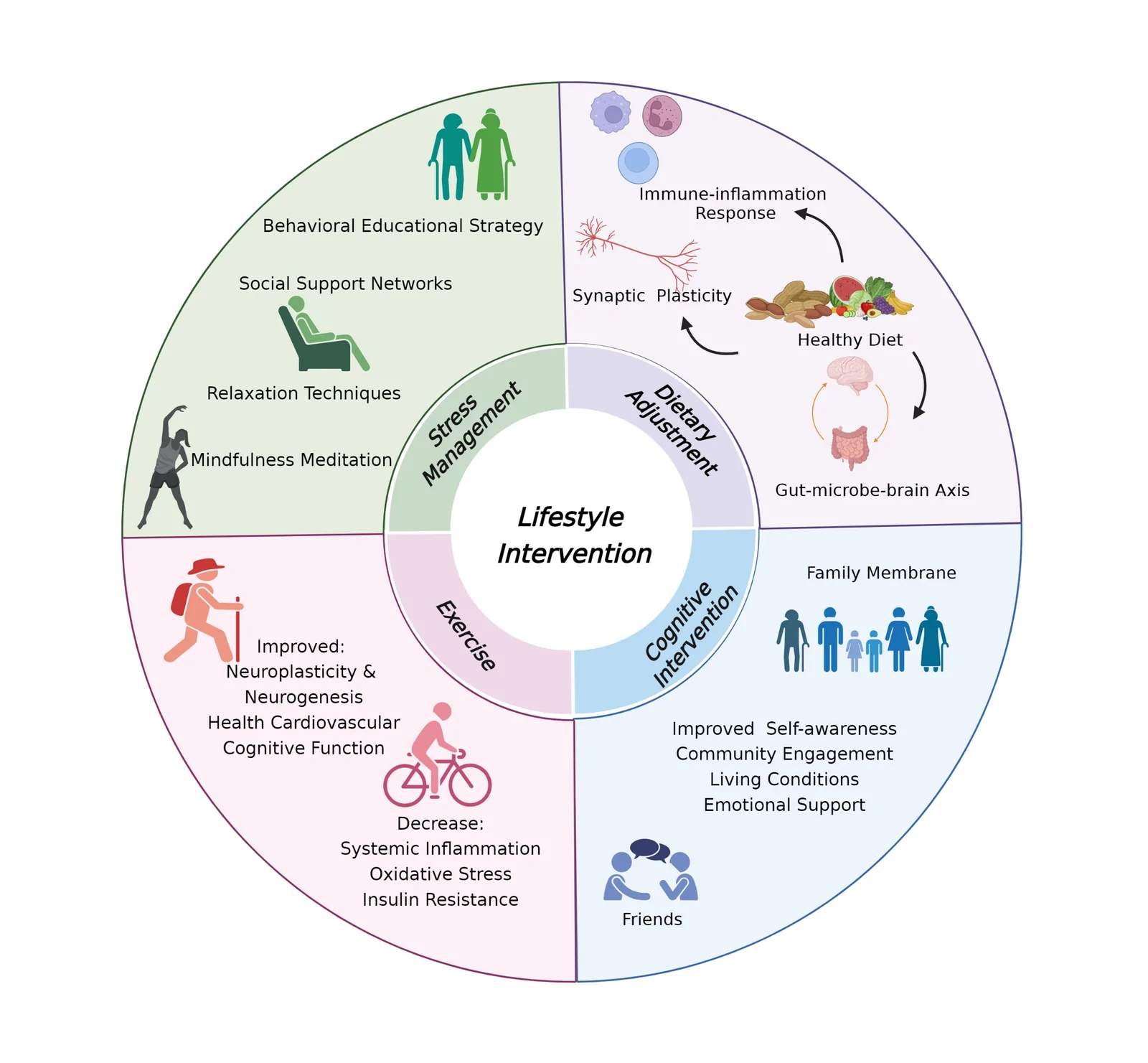
Components of healthy aging.

Lifestyle factors play a pivotal role in modulating the aging process and influencing the onset and progression of age-associated diseases. Extensive research has underscored the deep impact of lifestyle choices, encompassing exercise, diet, and stress management, on promoting healthy aging and mitigating the burden of age-related ailments. Figure 1 shows all the components of lifestyle interventions required as a part of non-pharmacological approaches.

#### 2.1.1. Impact of Lifestyle Factors on Aging and Age-Associated Diseases

The interplay between lifestyle and aging is intricate, with lifestyle choices exerting both direct and indirect effects on cellular aging processes and the development of age-related diseases. Sedentary behavior, poor dietary habits, and chronic stress have been implicated as major contributors to accelerated aging and heightened susceptibility to age-associated ailments [2]. Conversely, adopting a healthful lifestyle characterized by regular physical activity, balanced nutrition, and effective stress management has been associated with enhanced longevity and reduced risk of chronic diseases [6].

#### 2.1.2. Role of Exercise, Diet, and Stress Management in Promoting Healthy Aging

##### 1) Exercise:

Regular physical activity is a cornerstone of healthy aging, exerting multifaceted benefits on physiological function and overall well-being. Aerobic exercise enhances cardiovascular health, improves muscle strength and endurance, and promotes cognitive function by stimulating neurogenesis and synaptic plasticity [7]. Moreover, exercise attenuates systemic inflammation, oxidative stress, and insulin resistance, thereby mitigating the risk of age-related conditions such as cardiovascular disease, diabetes, and cognitive decline [8].

##### 2) Diet:

Dietary patterns significantly influence the aging process and disease susceptibility, with mounting evidence highlighting the role of nutrition in promoting healthy aging. A diet rich in fruits, vegetables, whole grains, and lean proteins provides essential nutrients, antioxidants, and bioactive compounds that confer protection against cellular damage and chronic inflammation [4, 9]. Conversely, excessive consumption of processed foods, saturated fats, and sugary beverages exacerbates oxidative stress, inflammation, and metabolic dysfunction, predisposing individuals to obesity, diabetes, and cardiovascular disease [10].

##### 3)Stress Management:

Chronic stress accelerates cellular aging and heightens vulnerability to age-associated diseases through dysregulation of the hypothalamic-pituitary-adrenal (HPA) axis and dysregulation of the immune system [11]. Adopting stress-reducing strategies such as mindfulness meditation, relaxation techniques, and social support networks mitigates the deleterious effects of stress on health outcomes and promotes resilience in the face of adversity [12].

A longitudinal study demonstrated that individuals engaging in regular physical activity exhibited slower rates of cognitive decline and reduced the risk of developing dementia compared to sedentary counterparts [13]. The landmark Mediterranean diet intervention trial revealed that adherence to some Mediterranean dietary patterns rich in olive oil, fruits, vegetables, and nuts reduced the incidence of cardiovascular events by 30% in high-risk individuals [14]. A randomized controlled trial [15] demonstrated that participation in a mindfulness-based stress reduction program significantly attenuated symptoms of depression and anxiety and improved psychological well-being among older adults [16]. Lifestyle interventions comprising exercise, diet, and stress management represent potent strategies for promoting healthy aging and ameliorating the burden of age-associated diseases. By adopting a proactive approach to lifestyle modification, individuals can optimize their health span and enhance their resilience to the challenges of aging.

### 2.2. Cognitive Interventions

Non-pharmacological approaches to supporting cognitive health in aging are gaining traction as effective strategies to mitigate cognitive decline and enhance cognitive resilience. Cognitive interventions encompass a spectrum of techniques ranging from cognitive training programs to mindfulness practices and other mental health strategies, all aimed at preserving cognitive function and promoting psychological well-being in older adults.

#### 2.2.1. Non-pharmacological Approaches to Supporting Cognitive Health

As individuals age, cognitive function naturally changes, with declines observed in various domains such as memory, processing speed, and executive function. Non-pharmacological interventions offer promising avenues for attenuating age-related cognitive decline and optimizing cognitive performance through targeted cognitive training, mindfulness-based practices, and psychological interventions.

##### 2.2.1.1. Cognitive training

Cognitive training programs involve structured exercises designed to enhance specific cognitive abilities such as memory, attention, and problem-solving skills. These interventions typically employ techniques such as mnemonic strategies, mental imagery, and task-specific practice to improve cognitive function. Meta-analytic studies have demonstrated that cognitive training interventions yield modest but significant improvements in cognitive performance, with notable benefits observed in older adults with mild cognitive impairment [17].

##### 2.2.1.2. Mindfulness and other mental health strategies

Mindfulness-based interventions encompass mindfulness meditation, yoga, and other contemplative practices aimed at cultivating present-moment awareness and emotional regulation. Emerging evidence suggests that regular mindfulness practice mitigates cognitive decline by reducing stress, enhancing attentional control, and fostering neuroplasticity in brain regions implicated in cognitive processing [18, 19]. Additionally, psychological interventions such as cognitive-behavioral therapy (CBT) and psychoeducation empower individuals to challenge negative thought patterns, cope with stressors, and enhance psychological resilience, thereby strengthening cognitive well-being in aging populations [20].

A randomized controlled trial demonstrated that a multi-domain cognitive training program incorporating memory, attention, and executive function exercises led to significant improvements in cognitive performance and functional independence among older adults with subjective cognitive decline [21]. Longitudinal studies [18, 22] reported that engagement in mindfulness-based interventions was associated with preserved cognitive function and reduced risk of cognitive decline in older adults over time. Meta-analyses [17, 20] provided robust evidence supporting the efficacy of cognitive training and psychological interventions in enhancing cognitive function and psychological well-being in aging populations. Non-pharmacological interventions targeting cognitive health offer promising avenues for preserving cognitive function and enhancing psychological resilience in aging populations. By integrating cognitive training, mindfulness practices, and psychological interventions into comprehensive cognitive health programs, individuals can optimize cognitive performance and maintain cognitive vitality as they age.

### 2.3. Social and Environmental Factors

Investigating the influence of social connections and environmental factors on aging unveils crucial insights into strategies for promoting healthy aging and mitigating the impact of age-related challenges. Understanding the significance of social support, community engagement, and living conditions sheds light on effective interventions aimed at fostering social well-being and optimizing health outcomes in older adults.

#### 2.3.1. Influence of Social Connections and Environmental Factors on Aging

Social connections and environmental factors exert profound effects on the aging process, influencing physical health, cognitive function, and psychological well-being. Robust social networks and supportive relationships serve as protective factors against loneliness, depression, and cognitive decline, buffering individuals from the adverse effects of stress and isolation [23]. Conversely, social isolation and environmental stressors such as pollution, inadequate housing, and neighborhood crime pose significant risks to health and well-being in aging populations, exacerbating chronic diseases, and impairing quality of life [24].

#### 2.3.2. Importance of Social Support, Community Engagement, and Living Conditions

##### 1)Social Support:

Social support encompasses emotional, instrumental, and informational assistance provided by social networks, family members, and community resources. Strong social support networks promote resilience in the face of adversity, enhance coping mechanisms, and foster a sense of belonging and connectedness, thereby mitigating the negative effects of stress and improving overall health outcomes [25].

##### 2)Community Engagement:

Active participation in community activities, social clubs, and volunteer organizations fosters social integration, promotes cognitive stimulation, and enhances psychosocial well-being in older adults [26]. Engaging with peers and contributing to meaningful activities not only provides a sense of purpose and fulfillment but also cultivates a supportive social environment conducive to healthy aging.

##### 3)Living Conditions:

Access to safe and supportive living environments is paramount for promoting health and well-being in older adults. Adequate housing, neighborhood amenities, and transportation services facilitate social interaction, access to healthcare, and engagement in recreational activities, thereby enhancing the quality of life and promoting independence in aging populations [27].

The Village model, a community-based aging-in-place initiative, provides older adults with access to supportive services, social activities, and volunteer assistance, enabling them to age in their own homes while remaining connected to their communities [28]. The Experience Corps program engages older adults as volunteer tutors and mentors in public schools, promoting intergenerational relationships, enhancing cognitive function, and fostering a sense of purpose and social contribution [29]. The Naturally Occurring Retirement Community (NORC) Supportive Services Program offers comprehensive support services and social activities to residents of age-integrated housing complexes, facilitating aging in place and promoting social connectedness and well-being [30].

**Figure 2.**
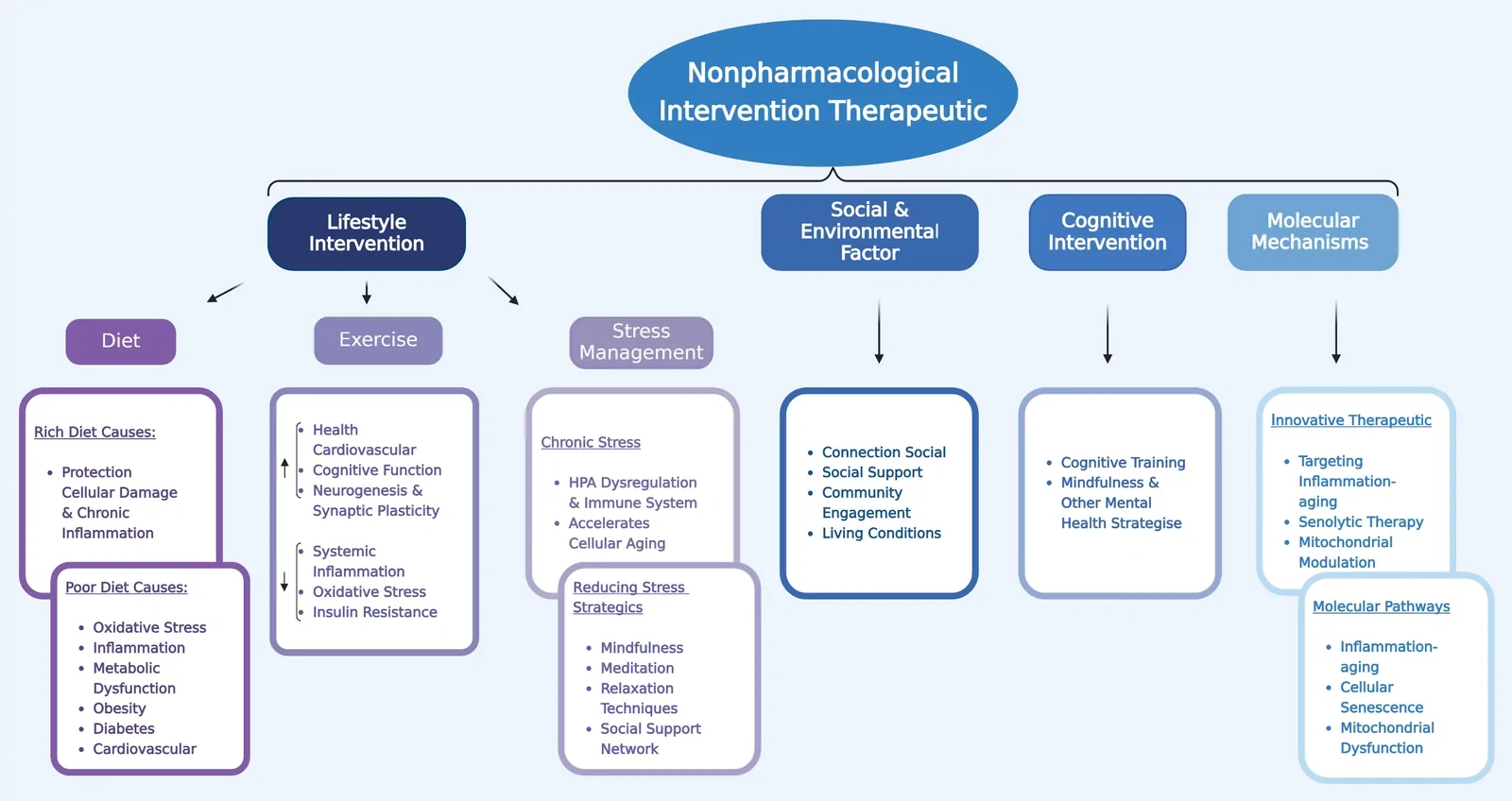
**Possible interventions and treatments against aging-related diseases**. Daily lifestyle changes, such as exercise, dietary interventions, stress management, also social and environmental factors, cognitive intervention, and molecular Mechanisms including mitochondrial modulation, senolytic therapy can inhibit aging and reduce the occurrence and development of aging-related diseases, subsequently promoting healthy aging and longevity. nonpharmacological therapy is the main strategy targeting aging. Created with BioRender.com.

Understanding the influence of social and environmental factors on aging is instrumental in designing effective interventions to promote healthy aging and enhance the well-being of older adults. Figure 2 depicts a pictorial representation of the possible interventions and treatments against aging-related diseases. By fostering social support networks, promoting community engagement, and improving living conditions, society can create an enabling environment that empowers older adults to age with dignity, independence, and vitality.

## 3. New Insights into Molecular Mechanisms and Innovative Therapeutic Approaches for Aging-Related Diseases

### 3.1. Molecular Mechanisms

Recent advancements in understanding the molecular basis of aging-related diseases have provided invaluable insights into the intricate interplay of molecular pathways and cellular processes underlying age-associated pathologies. By elucidating key molecular mechanisms, researchers have identified novel therapeutic targets and innovative strategies for combating aging-related diseases and promoting healthy aging.

#### 3.1.1. Recent Advancements in Understanding the Molecular Basis

Advances in molecular biology, genomics, and systems biology have revolutionized our understanding of the molecular mechanisms driving aging-related diseases. High-throughput sequencing technologies, genome-wide association studies (GWAS), and functional genomics approaches have facilitated the identification of genetic variants, epigenetic modifications, and molecular signatures associated with age-related pathologies, providing a comprehensive framework for dissecting disease etiology and progression [2].

#### 3.1.2. Key Molecular Pathways and Cellular Processes Involved

##### 1)Inflamm-aging:

Chronic low-grade inflammation, termed "inflamm-aging," emerges as a hallmark feature of aging and contributes to the pathogenesis of age-related diseases such as Alzheimer's disease, cardiovascular disease, and cancer. Dysregulation of inflammatory signaling pathways, including the nuclear factor-kappa B (NF-κB) pathway and the inflammasome, fuels a pro-inflammatory milieu characterized by elevated levels of cytokines, chemokines, and reactive oxygen species, perpetuating tissue damage and promoting disease progression [31].

##### 2)Cellular senescence:

Cellular senescence, a state of irreversible cell cycle arrest, plays a dual role in aging and age-related diseases. While senescent cells contribute to tissue remodeling and wound healing in the short term, their accumulation over time promotes tissue dysfunction, chronic inflammation, and tumor progression. Senescent cells secrete a plethora of bioactive molecules, collectively termed the senescence-associated secretory phenotype (SASP), which exert paracrine effects on neighboring cells, exacerbating tissue dysfunction and contributing to the pathogenesis of age-related disorders [32].

**Figure 3.**
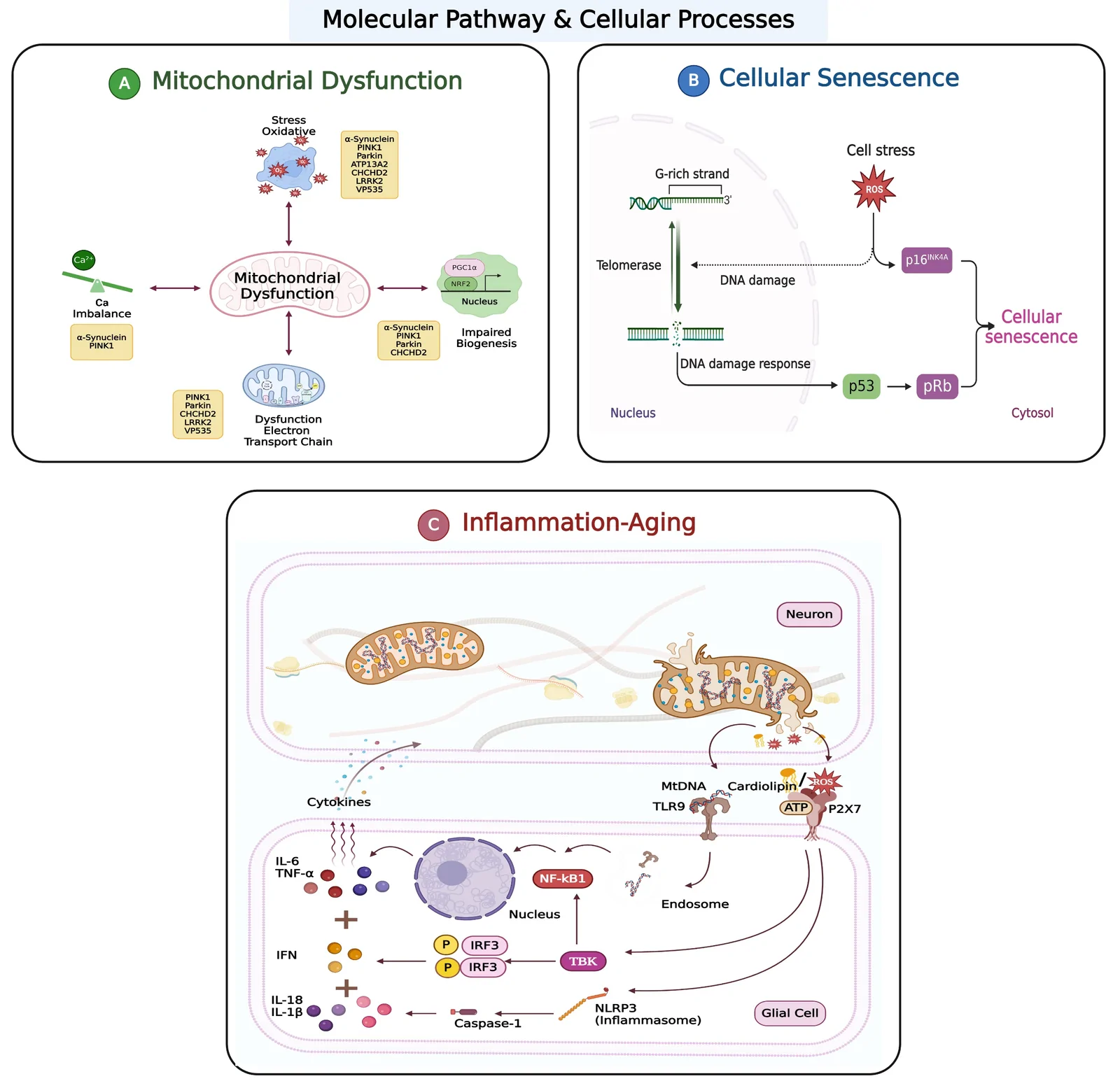
**Molecular mechanisms aging diseases-related**. (**A**) Mitochondrial dysfunction causes increase of stress oxidative, impaired biogenesis, electron transport chain, and in Ca+2balance, and change expression of disease markers. (**B**) Induce of cell stress, causes DNA damage and dysfunction of gene expression and in led to cellular senescence. (**C**) Dysregulation of inflammatory signaling pathways, including the NF-κB pathway and inflammasome, creates a pro-inflammatory environment that increases the levels of cytokines, chemokines, and reactive oxygen species, causing tissue damage and the disease progresses. NF-κB: nuclear factor-kappa B, NLRP3: nucleotide-binding domain, leucine-rich-containing family, pyrin domain-containing-3, IRF3: Interferon regulatory factor 3, IFN; Interferon, TNF-α: Tumor Necrosis Factor-alpha, IL: Interleukin, TBK: TANK-binding kinase, TLR-9: Toll-like receptor 9, MtDNA: mitochondrial DNA, Created with BioRender.com.

##### 3)Mitochondrial dysfunction:

Mitochondrial dysfunction emerges as a central feature of aging, driving cellular energy depletion, oxidative stress, and impaired bioenergetics. Accumulation of mitochondrial DNA (mtDNA) mutations, impaired mitochondrial dynamics, and dysregulated mitochondrial quality control mechanisms compromise mitochondrial function, rendering cells susceptible to oxidative damage and metabolic dysfunction, thereby predisposing individuals to age-related diseases such as neurodegenerative disorders and metabolic syndrome [5]. Figure 3 illustrates the molecular mechanisms underlying aging-related diseases, including mitochondrial dysfunction, cell stress induction, and dysregulation of inflammatory signaling pathways.

#### 3.1.3. Innovative Therapeutic Approaches

##### 1)Targeting inflamm-aging:

Anti-inflammatory interventions targeting key mediators of inflamm-aging, such as NF-κB inhibitors, interleukin-1 (IL-1) antagonists, and senolytic agents, hold promise for attenuating chronic inflammation and mitigating the progression of age-related diseases [33].

##### 2)Senolytic therapy:

Senolytic drugs selectively eliminate senescent cells, thereby alleviating senescence-associated inflammation and tissue dysfunction. Preclinical studies have demonstrated the efficacy of senolytic agents, such as dasatinib and quercetin, in improving health span and ameliorating age-related phenotypes in animal models [34].

##### 3)Mitochondrial modulation:

Strategies aimed at restoring mitochondrial function and mitigating oxidative stress, such as mitochondrial-targeted antioxidants, mitochondrial biogenesis inducers, and mitophagy enhancers, offer potential therapeutic avenues for combating mitochondrial dysfunction and age-related diseases [35].

Recent insights into the molecular mechanisms of aging-related diseases have paved the way for the development of innovative therapeutic approaches aimed at targeting key molecular pathways and cellular processes implicated in disease pathogenesis. By harnessing the power of molecular interventions, researchers aim to revolutionize the landscape of aging research and usher in a new era of precision medicine for promoting healthy aging and combating age-related diseases.

### 3.2. Innovative Therapeutic Approaches

Emerging therapeutic strategies for aging-related diseases represent a paradigm shift in healthcare, offering novel avenues for personalized treatment and precision medicine. From gene therapy and stem cell interventions to targeted pharmacotherapy and immunomodulation, innovative approaches hold promise for revolutionizing disease management and improving outcomes in aging populations.

#### 3.2.1. Exploring Emerging Therapeutic Strategies

In the quest to combat aging-related diseases, researchers are exploring a diverse array of innovative therapeutic modalities that target underlying molecular mechanisms and cellular pathways implicated in disease pathogenesis. These approaches encompass precision medicine, gene therapy, regenerative medicine, and immunomodulatory interventions, aimed at tailoring treatment strategies to individual patient profiles and disease characteristics [3].

#### 3.2.2. Role of Personalized Medicine, Gene Therapy, and Other Innovative Interventions

##### 1)Personalized medicine:

Personalized medicine leverages advances in genomics, proteomics, and bioinformatics to tailor medical interventions to the unique genetic makeup and physiological characteristics of individual patients. By integrating genomic data, biomarker profiling, and clinical phenotyping, personalized medicine enables clinicians to optimize treatment selection, dosage regimens, and therapeutic strategies, thereby maximizing therapeutic efficacy and minimizing adverse effects [36].

##### 2)Gene therapy:

Gene therapy holds promise for treating aging-related diseases by correcting genetic defects, modulating gene expression, and enhancing cellular function. Advancements in viral vectors, genome editing technologies, and nucleic acid delivery systems have facilitated the development of gene-based therapeutics targeting a wide spectrum of age-related disorders, including inherited genetic disorders, neurodegenerative diseases, and cardiovascular conditions [37].

##### 3)Regenerative medicine:

Regenerative medicine encompasses a suite of innovative approaches aimed at harnessing the regenerative potential of stem cells, tissue engineering, and biomaterials to repair, replace, or regenerate damaged tissues and organs. Stem cell therapies, organoid cultures, and tissue engineering techniques offer promising avenues for restoring tissue function and promoting tissue regeneration in aging-related diseases such as Parkinson's disease, osteoarthritis, and age-related macular degeneration [38].

**Figure 4.**
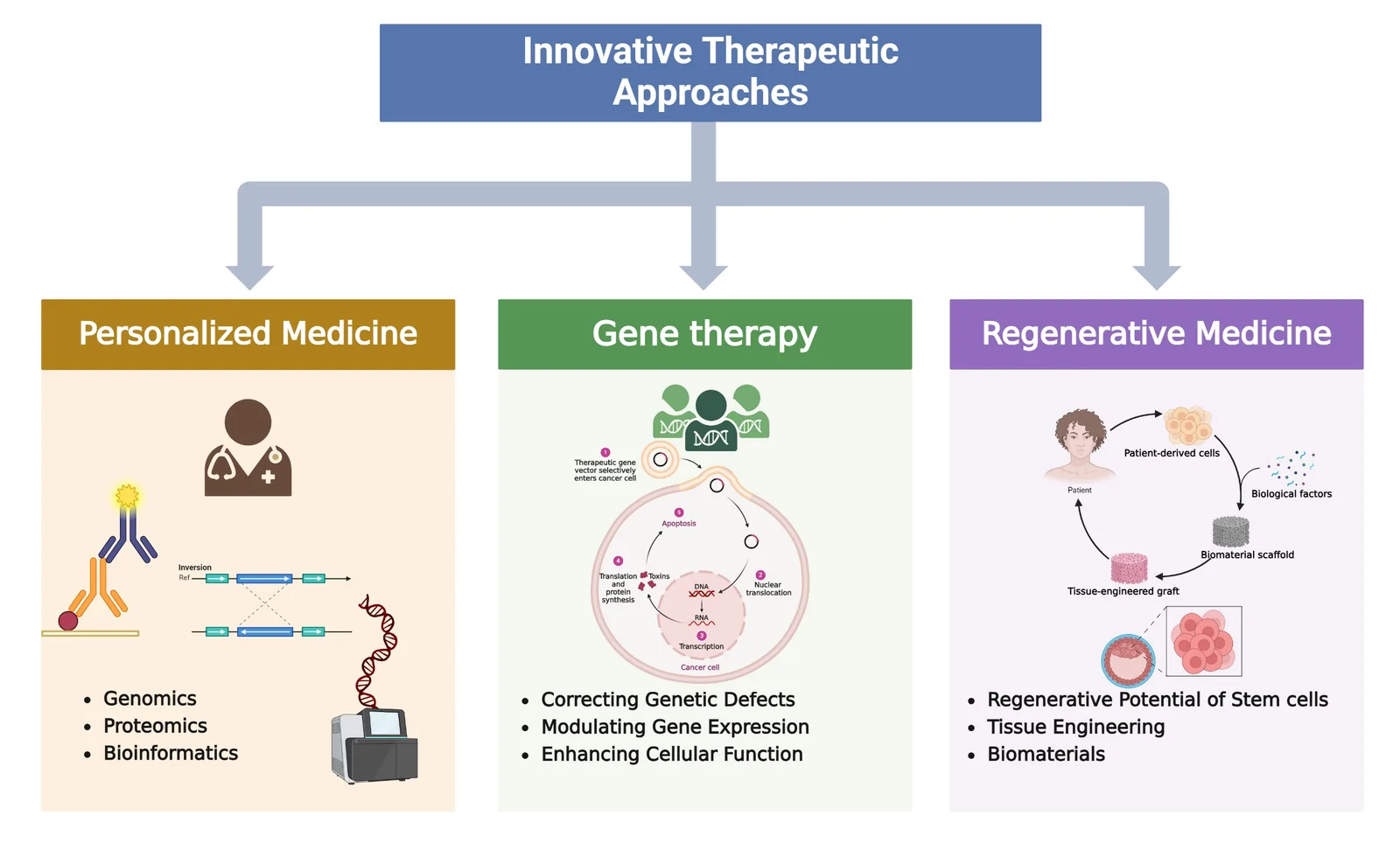
Innovative therapeutic strategies for aging-related diseases.

A phase I/II clinical trial by Barthélémy and Wein (2018) [39] demonstrated the safety and efficacy of a personalized gene therapy approach targeting the underlying genetic mutations in patients with Duchenne muscular dystrophy, paving the way for personalized gene-based treatments in rare genetic disorders. The ongoing Precision Medicine Initiative led by the National Institutes of Health aims to advance personalized medicine by integrating genomics, electronic health records, and longitudinal data to tailor prevention and treatment strategies to individual patient characteristics and disease trajectories [40]. A phase II clinical trial [41] explored the safety and efficacy of autologous mesenchymal stem cell transplantation in patients with moderate-to-severe knee osteoarthritis, demonstrating improvements in pain, function, and joint structure over a 12-month follow-up period. Figure 4 depicts the innovative strategies including personalized medication, gene therapy and regenerative medication. The innovative therapeutic approaches hold immense promise for revolutionizing disease management and improving outcomes in aging-related diseases. By harnessing the power of personalized medicine, gene therapy, and regenerative medicine, researchers aim to usher in a new era of precision medicine that transforms the landscape of aging research and clinical practice.

### 3.3. Interdisciplinary Approaches

Emphasizing the importance of interdisciplinary collaboration in addressing aging-related diseases underscores the multifaceted nature of these complex health challenges and the need for comprehensive, integrated approaches to disease prevention, diagnosis, and treatment. By bridging the gap between diverse fields of research and clinical practice, interdisciplinary collaboration fosters innovation, facilitates knowledge exchange, and enhances the development of more effective interventions for promoting healthy aging and combating age-related diseases.

#### 3.3.1. Importance of Interdisciplinary Collaboration

Interdisciplinary collaboration plays a pivotal role in advancing our understanding of aging-related diseases and translating scientific discoveries into tangible clinical applications. By bringing together experts from diverse disciplines such as biology, medicine, genetics, epidemiology, psychology, sociology, and engineering, interdisciplinary research teams can leverage complementary expertise, perspectives, and methodologies to address complex scientific questions and clinical challenges in aging research [42].

#### 3.3.2. Integrating Knowledge from Different Fields

Integrating knowledge from different fields offers a holistic approach to addressing the multifactorial nature of aging-related diseases, encompassing genetic, environmental, social, and behavioral determinants of health. By synthesizing insights from basic science, clinical research, population health, and health policy, interdisciplinary approaches can elucidate the interconnectedness of biological, psychological, and social factors contributing to disease pathogenesis and identify novel intervention targets and strategies for disease prevention and management [43].

The Alzheimer's Disease Neuroimaging Initiative (ADNI), a collaborative research effort involving neuroscientists, radiologists, geneticists, and bioinformaticians, aims to identify biomarkers of Alzheimer's disease progression, develop predictive models for early diagnosis, and accelerate the discovery of novel therapeutic targets through multi-modal neuroimaging, genomics, and systems biology approaches [44, 45]. The Longevity Genomics Consortium (LGC), comprising researchers from diverse disciplines including genetics, epidemiology, and bioinformatics, collaborates to identify genetic variants associated with healthy aging and longevity. By integrating data from genome-wide association studies (GWAS), transcriptomics, and functional genomics, the LGC aims to unravel the genetic determinants of successful aging and identify druggable targets for age-related diseases [46]. The Health and Retirement Study (HRS), a longitudinal study funded by the National Institute on Aging, integrates insights from sociology, economics, epidemiology, and public health to examine the social, economic, and behavioral determinants of health and aging. By collecting comprehensive data on health status, cognitive function, socioeconomic factors, and healthcare utilization, the HRS provides valuable insights into the complex interplay of individual and contextual factors shaping health outcomes in aging populations [47]. Interdisciplinary collaboration is essential for advancing aging research and developing effective interventions for addressing aging-related diseases. By fostering collaboration across disciplines, researchers can harness the collective expertise and resources needed to tackle the multifaceted challenges of aging and promote healthy aging for all.

## 4. Challenges and Future Directions

### 4.1. Identifying Current Challenges in the Field of Aging-Related Diseases

The field of aging-related diseases faces numerous challenges that impede progress in understanding disease mechanisms, developing effective interventions, and improving patient outcomes. Some key challenges include the complexity of age-related pathologies, limited translational research efforts, disparities in access to healthcare and resources, and the need for interdisciplinary collaboration and funding support [3].

### 4.2. Discussing Potential Obstacles to Implementing Non-pharmacological and Innovative Approaches

Despite the promise of non-pharmacological and innovative approaches in addressing aging-related diseases, several obstacles hinder their widespread implementation and adoption. These obstacles include insufficient evidence-based guidelines, limited healthcare provider knowledge and training, patient adherence and motivation issues, financial barriers, and regulatory challenges associated with novel therapies and interventions [48].

### 4.3. Providing Insights into Future Research Directions and Opportunities

Future research in the field of aging-related diseases should focus on several key areas to overcome current challenges and advance scientific knowledge and clinical practice. These areas include:

#### 4.3.1. Precision medicine and personalized interventions

Leveraging advances in genomics, biomarker discovery, and digital health technologies to tailor interventions to individual patient profiles and disease characteristics, thereby optimizing treatment efficacy and minimizing adverse effects.

#### 4.3.2. Interdisciplinary collaboration and translational research

Promoting collaboration across diverse disciplines and fostering translational research efforts to bridge the gap between basic science discoveries and clinical applications, accelerating the development and implementation of novel therapeutic strategies

#### 4.3.3. Health equity and access to care

Addressing disparities in healthcare access, resources, and outcomes among underserved populations, particularly marginalized and vulnerable groups, through community-based interventions, health policy initiatives, and advocacy efforts

#### 4.3.4. Patient-centered care and behavioral interventions

Incorporating patient preferences, values, and goals into treatment decision-making and developing innovative behavioral interventions to promote healthy lifestyle behaviors, enhance treatment adherence, and improve patient outcomes

#### 4.3.5. Regulatory and policy reform: Advocating for regulatory and policy reforms to streamline the approval process for novel therapies and interventions, foster innovation, and ensure equitable access to safe and effective treatments for aging-related diseases

Addressing the challenges and opportunities in the field of aging-related diseases requires a concerted effort from researchers, healthcare providers, policymakers, and stakeholders. By embracing interdisciplinary collaboration, promoting patient-centered care, and advocating for health equity and policy reform, we can pave the way for transformative advancements in aging research and clinical practice, ultimately improving the health and well-being of aging populations worldwide.

## 5. Conclusion

This study provides an extensive analysis of aging-related diseases, emphasizing the imperative to comprehending their underlying molecular mechanisms and investigating novel therapeutic approaches. It emphasizes the significance of non-pharmacological interventions alongside conventional treatments, highlighting the potential of lifestyle modifications, cognitive interventions, and social engagement in facilitating healthy aging. Moreover, the study underscores the emergence of innovative therapeutic strategies such as gene therapy and precision medicine, presenting promising avenues for personalized treatment and precision healthcare in aging-related diseases. Despite advancements, challenges persist, including the complexity of age-related pathologies and disparities in healthcare access. Looking forward, sustained research, collaboration, and advocacy efforts are crucial for advancing scientific knowledge, translating findings into clinical applications, and ensuring equitable access to innovative treatments for aging-related diseases. Through collective efforts, we can navigate the intricacies of aging, enhance health outcomes, and work towards a future where aging is characterized by vitality, dignity, and well-being globally.

## References

[1] Niccoli T, Partridge L (2012). Aging as a risk factor for disease. Curr Biol, 22:R741-R752. 10.1016/j.cub.2012.07.024 22975005

[2] López-Otín C, Blasco MA, Partridge L, Serrano M, Kroemer G (2013). The hallmarks of aging. Cell, 153:1194-1217. 10.1016/j.cell.2013.05.039 PMC3836174 23746838

[3] Kennedy BK, Berger SL, Brunet A, Campisi J, Cuervo AM, Epel ES, Sierra F (2014). Geroscience: linking aging to chronic disease. Cell, 159:709-713. 10.1016/j.cell.2014.10.039 PMC4852871 25417146

[4] Fontana L, Partridge L, Longo VD (2010). Extending healthy life span from yeast to humans. Science, 328:321-326. 10.1126/science.1172539 PMC3607354 20395504

[5] López-Otín C, Galluzzi L, Freije JM, Madeo F, Kroemer G (2016). Metabolic control of longevity. Cell, 166:802-821. 10.1016/j.cell.2016.07.031 27518560

[6] Booth FW, Roberts CK, Laye MJ (2012). Lack of exercise is a major cause of chronic diseases. Compr Physiol, 2:1143-1211. 10.1002/cphy.c110025 PMC4241367 23798298

[7] Laurin D, Verreault R, Lindsay J, MacPherson K, Rockwood K (2001). Physical activity and risk of cognitive impairment and dementia in elderly persons. Arch Neurol, 58:498-504. 10.1001/archneur.58.3.498 11255456

[8] Nagamatsu LS, Handy TC, Hsu CL, Voss M, Liu-Ambrose T (2012). Resistance training promotes cognitive and functional brain plasticity in seniors with probable mild cognitive impairment. Arch Intern Med, 172:666-668. 10.1001/archinternmed.2012.379 PMC3514552 22529236

[9] Bansal V, Chatterjee I (2022). Association of vitamins and neurotransmitters: understanding the effect on schizophrenia. Neurochem J, 16:39-45.

[10] Mozaffarian D, Hao T, Rimm EB, Willett WC, Hu FB (2011). Changes in diet and lifestyle and long-term weight gain in women and men. N Engl J Med, 364:2392-2404. 10.1056/NEJMoa1014296 PMC3151731 21696306

[11] Epel ES, Blackburn EH, Lin J, Dhabhar FS, Adler NE, Morrow JD, Cawthon RM (2004). Accelerated telomere shortening in response to life stress. Proc Natl Acad Sci, 101:17312-17315. 10.1073/pnas.0407162101 PMC534658 15574496

[12] Black DS, O’Reilly GA, Olmstead R, Breen EC, Irwin MR (2015). Mindfulness meditation and improvement in sleep quality and daytime impairment among older adults with sleep disturbances: a randomized clinical trial. JAMA Intern Med, 175:494-501. 10.1001/jamainternmed.2014.8081 PMC4407465 25686304

[13] Lee AT, Richards M, Chan WC, Chiu HF, Lee RS, Lam LC (2018). Association of daily intellectual activities with lower risk of incident dementia among older Chinese adults. JAMA psychiat, 75:697-703. 10.1001/jamapsychiatry.2018.0657 PMC6583858 29847678

[14] Estruch R, Ros E, Salas-Salvadó J, Covas MI, Corella D, Arós F, Martínez-González MA (2013). Primary prevention of cardiovascular disease with a Mediterranean diet. N Engl J Med, 368:1279-1290.

[15] Carlson EN (2013). Overcoming the barriers to self-knowledge: Mindfulness as a path to seeing yourself as you really are. Perspect Psychol Sci, 8:173-186. 10.1177/1745691612462584 26172498

[16] Chatterjee I, Chatterjee S (2023). Investigating the symptomatic and morphological changes in the brain based on pre and post-treatment: a critical review from clinical to neuroimaging studies on schizophrenia. IBRO Neurosci Rep, 14:366-374. 10.1016/j.ibneur.2023.03.008 PMC10300448 37388500

[17] Gates N, Valenzuela M, Sachdev PS, Fiatarone Singh MA (2014). Psychological well-being in individuals with mild cognitive impairment. Clin Interv Aging, 9:779-792. 10.2147/CIA.S58866 PMC4020883 24855347

[18] Gard DE, Sanchez AH, Starr J, Cooper S, Fisher M, Rowlands A, Vinogradov S (2014). Using self-determination theory to understand motivation deficits in schizophrenia: the ‘why’of motivated behavior. Schizophr Res, 156:217-222. 10.1016/j.schres.2014.04.027 PMC4084414 24853060

[19] Chatterjee I, Kumar V, Rana B, Agarwal M, Kumar N (2020). Impact of aging on the brain regions of the schizophrenia patients: an fMRI study using evolutionary approach. Multimed Tools Appl, 79:24757-24779.

[20] Hedman-Lagerlöf M, Hedman-Lagerlöf E, Öst LG (2018). The empirical support for mindfulness-based interventions for common psychiatric disorders: a systematic review and meta-analysis. Psychol Med, 48:2116-2129. 10.1017/S0033291718000259 29455695

[21] Belleville S, Moussard A, Ansaldo AI, Belchior P, Bherer L, Bier N, Anderson ND (2019). Rationale and protocol of the ENGAGE study: a double-blind randomized controlled preference trial using a comprehensive cohort design to measure the effect of a cognitive and leisure-based intervention in older adults with a memory complaint. Trials, 20:1-18. 10.1186/s13063-019-3250-6 PMC6532200 31118095

[22] Chételat G, Landeau B, Eustache F, Mézenge F, Viader F, de La Sayette V, Baron JC (2005). Using voxel-based morphometry to map the structural changes associated with rapid conversion in MCI: a longitudinal MRI study. Neuroimage, 27:934-946. 10.1016/j.neuroimage.2005.05.015 15979341

[23] Holt-Lunstad J, Smith TB, Layton JB (2010). Social relationships and mortality risk: a meta-analytic review. PLoS Med, 7:1000316. 10.1371/journal.pmed.1000316 PMC2910600 20668659

[24] Evans GW (2003). The built environment and mental health. J Urban Health, 80:536-555. 10.1093/jurban/jtg063 PMC3456225 14709704

[25] Uchino BN (2006). Social support and health: a review of physiological processes potentially underlying links to disease outcomes. J Behav Med, 29:377-387. 10.1007/s10865-006-9056-5 16758315

[26] Pillemer K, Fuller-Rowell TE, Reid MA, Wells NM (2010). Environmental volunteering and health outcomes over a 20-year period. Gerontologist, 50:594-602. 10.1093/geront/gnq007 PMC2937248 20172902

[27] Balfour JL, Kaplan GA (2002). Neighborhood environment and loss of physical function in older adults: evidence from the Alameda County Study. Am J Epidemiol, 155:507-515. 10.1093/aje/155.6.507 11882524

[28] Scharlach A, Graham C, Lehning A (2012). The “Village” model: A consumer-driven approach for aging in place. Gerontologist, 52:418-427. 10.1093/geront/gnr083 21873280

[29] Fried LP, Carlson MC, Freedman M, Frick KD, Glass TA, Hill J, Zeger S (2004). A social model for health promotion for an aging population: initial evidence on the Experience Corps model. J Urban Health, 81:64-78. 10.1093/jurban/jth094 PMC3456134 15047786

[30] Gruenewald TL, Tanner EK, Fried LP, Carlson MC, Xue QL, Parisi JM, Seeman TE (2016). The Baltimore Experience Corps Trial: Enhancing generativity via intergenerational activity engagement in later life. J Gerontol B Psychol Sci Soc Sci, 71:661-670. 10.1093/geronb/gbv005 PMC4903034 25721053

[31] Franceschi C, Garagnani P, Parini P, Giuliani C, Santoro A (2018). Inflammaging: a new immune-metabolic viewpoint for age-related diseases. Nat Rev Endocrinol, 14:576-590. 10.1038/s41574-018-0059-4 30046148

[32] Childs BG, Durik M, Baker DJ, van Deursen JM (2015). Cellular senescence in aging and age-related disease: from mechanisms to therapy. Nat Med, 21:1424-1435. 10.1038/nm.4000 PMC4748967 26646499

[33] Fulop T, Larbi A, Dupuis G, Le Page A, Frost EH, Cohen AA, Franceschi C (2018). Immunosenescence and inflamm-aging as two sides of the same coin: friends or foes?. Front Immunol, 8:328099. 10.3389/fimmu.2017.01960 PMC5767595 29375577

[34] Kirkland JL, Tchkonia T (2020). Senolytic drugs: from discovery to translation. J Intern Med, 288:518-536. 10.1111/joim.13141 PMC7405395 32686219

[35] Houtkooper RH, Pirinen E, Auwerx J (2012). Sirtuins as regulators of metabolism and healthspan. Nat Rev Mol Cell Biol, 13:225-238. 10.1038/nrm3293 PMC4872805 22395773

[36] Jameson JL, Longo DL (2015). Precision medicine personalized, problematic, and promising. Obstet Gynecol Surv, 70:612-614. 10.1056/NEJMsb1503104 26014593

[37] Naldini L (2015). Gene therapy returns to centre stage. Nat, 526:351-360. 10.1038/nature15818 26469046

[38] Atala A, Lanza R, Mikos T, Nerem R (2018). Principles of regenerative medicine. Academic Press.

[39] Barthélémy F, Wein N (2018). Personalized gene and cell therapy for duchenne muscular dystrophy. Neuromuscul Disord, 28:803-824. 10.1016/j.nmd.2018.06.009 30224293

[40] Collins FS, Varmus H (2015). A new initiative on precision medicine. N Engl J Med, 372:793-795. 10.1056/NEJMp1500523 PMC5101938 25635347

[41] Lanza R, Gearhart J, Hogan B, Melton D, Pedersen R, Thomas ED, Thomson JA (2005). Essentials of stem cell biology. Elsevier. Academic Press.

[42] Sturmberg JP, Martin CM. (2013). Handbook of systems and complexity in health (pp. 1-17). New York: Springer.

[43] Burns-Hernandez LU, Greenberg JE (2011). Harvard MIT division of health sciences and technology. IEEE pulse, 2:68-69. 10.1109/MPUL.2011.941718 21791405

[44] Mueller SG, Weiner MW, Thal LJ, Petersen RC, Jack C, Jagust W, Beckett L (2005). The Alzheimer’s disease neuroimaging initiative. Neuroimaging Clin N Am, 15:869. 10.1016/j.nic.2005.09.008 PMC2376747 16443497

[45] Thakur M, Kumar A, Chatterjee I (2023). IoT-enabled solutions for Alzheimer's disease management: innovations and opportunities. Neurosci Res Notes, 6:255-261.

[46] Deelen J, Evans DS, Arking DE, Tesi N, Nygaard M, Liu X, Murabito JM (2019). A meta-analysis of genome-wide association studies identifies multiple longevity genes. Nat Commun, 10:3669. 10.1038/s41467-019-11558-2 PMC6694136 31413261

[47] Sonnega A, Faul JD, Ofstedal MB, Langa KM, Phillips JW, Weir DR (2014). Cohort profile: the health and retirement study (HRS). Int J Epidemiol, 43:576-585. 10.1093/ije/dyu067 PMC3997380 24671021

[48] Tao A, Ho KHM, Yang C, Chan HYL (2023). Effects of non-pharmacological interventions on psychological outcomes among older people with frailty: a systematic review and meta-analysis. Int J Nurs Stud, 140:104437. 10.1016/j.ijnurstu.2023.104437 36764033

